# Dynamic Indoor Localization Using Maximum Likelihood Particle Filtering

**DOI:** 10.3390/s21041090

**Published:** 2021-02-05

**Authors:** Wenxu Wang, Damián Marelli, Minyue Fu

**Affiliations:** 1 School of Automation, Guangdong University of Technology, Guangzhou 510006, China; wangwenxu0909@126.com (W.W.); minyue.fu@newcastle.edu.au (M.F.); 2 National Scientific and French Argentine International Center for Information and Systems Sciences, Technical Research Council, Rosario S2000, Argentina; 3 School of Electrical Engineering and Computer Science, University of Newcastle, Newcastle, NSW 2308, Australia

**Keywords:** indoor tracking, particle filter, channel state information, WiFi fingerprinting

## Abstract

A popular approach for solving the indoor dynamic localization problem based on WiFi measurements consists of using particle filtering. However, a drawback of this approach is that a very large number of particles are needed to achieve accurate results in real environments. The reason for this drawback is that, in this particular application, classical particle filtering wastes many unnecessary particles. To remedy this, we propose a novel particle filtering method which we call maximum likelihood particle filter (MLPF). The essential idea consists of combining the particle prediction and update steps into a single one in which all particles are efficiently used. This drastically reduces the number of particles, leading to numerically feasible algorithms with high accuracy. We provide experimental results, using real data, confirming our claim.

## 1. Introduction

Wireless positioning is the most popular approach for indoor location-based services. It finds applications in emergency rescue, smart home, security monitoring and many other areas. In contrast to outdoor positioning, whose dominant approach is based on the global positioning system (GPS), indoor positioning is hindered by multiple sources of interference and multipath effects in time-varying environments. In view of this, multiple techniques for indoor positioning are currently being investigated. The most popular ones are based on radio frequency identification [1,2], Bluetooth signals [3,4], ultra-wideband signals [5,6] and WiFi signals [7,8]. Due to their large coverage, low implementation cost and availability in existing mobile devices, WiFi-based indoor positioning is the most promising technique.

A broad classification of indoor localization methods can be done in two categories, namely, geometric mapping and fingerprinting [9]. In geometric mapping, the target’s position is estimated using geometric information like distances and angles from the target to a set of access points (APs) [10]. Typical techniques for obtaining geometric information are time of flight (ToF) [11] and angle of arrival (AoA) [12]. This approach typically requires obtaining information from more than three APs. the resulting methods are based on line of sight (LOS) signals, and therefore are easily influenced by obstacles. Strict synchronization is also needed between different devices. The above limitations are overcome by the fingerprinting method [13]. It consists of building a database containing the values of certain received signal features at a given set of known locations. Localization is then done by comparing the features obtained at the target’s location with those of the database. Localization based on fingerprinting is more immune to distortions caused by the environment, since the latter are coded in the feature database. However, it suffers from poor positioning accuracy when the target is in a position far from anyone in the database.

The most commonly WiFi signal feature used for indoor localization is its received signal strength (RSS) [14]. It measures the signal power strength at the receiver side. Although RSS measurements are easy to obtain, only a very rough estimation of the receiver’s position can be obtained from it. This is due to the fading and distortion suffered by signals propagating through multipaths. To tackle this problem, in recent years channel state information (CSI) has been used instead of RSS [9]. CSI contains information of the phase and amplitude value of each subcarrier of an orthogonal frequency division multiplexing (OFDM) channel. It can be readily obtained from any OFDM system based on the 802.11n protocol. Since multipath and fading information is represented in the CSI, its use for indoor localization leads to more accurate and stable results in comparison to RSS.

To improve the accuracy of any given localization method, extra information can be obtained from a motion model of the target. The common solution for fusing the information from the motion model with that from measurements consists of using some Bayesian tracking technique. We refer to resulting techniques as dynamic localization methods [15]. One such techniques is the Kalman filter [16,17,18]. While being optimal and having a closed-form, it only applies to linear Gaussian model. This assumption can be relaxed using the extended Kalman filter [19]. However, since this method is simply based on linearizing a non-linear model, it often leads to inaccurate results. This can be overcome by using particle filtering [20,21,22,23]. This essentially consists of approximating non-Gaussian probability distributions using a number of samples (particles) multiplied by their associated weights.

As it is known, a Bayesian tracking method consists of alternating two steps called prediction and update. In a particle filter, a set of new particles is drawn during each prediction step, whose weighs are determined during the subsequent update step. A drawback of this approach is that this weighting results in little use of those particles whose weights are very small. The number of wasted particles can be very large in the case where measurements already provide enough information to determine the target’s location with a reasonable accuracy, without using the information provided by the motion model. This is often the case in localization setups, for otherwise, algorithms not using a motion model would not work with reasonable accuracy. Consequently, a very large number of particles are required to avoid particle depletion after the update step, which would result in an inaccurate result. The vast number of required particles renders the approach numerically intractable in many applications.

In order to tackle the above shortcoming, a number of methods are available to reduce the estimation error resulting from using particle filtering with a restricted number of particles. In [21], a machine learning classifier is added to decide whether particles are in the correct room. In [22], a context variable, with values in the set {‘free space’, ‘constrained space’, ‘static space’}, is added to the state to account for the movement constraints at the target’s current position. Also, the authors of [23] divide the environment into reachable and unreachable areas, so as to discard particles falling into an unreachable area.

Generally speaking, the above methods aim to reduce the particle depletion phenomenon described above by complementing the motion model with environment- dependent information to decide where to draw particles. The aim of this work is to make the most of this principle, i.e., ideally to totally avoid particle depletion. To this end, we propose a novel particle filtering scheme which combines the prediction and update steps into a single one. In other words, we avoid drawing unnecessary particles during the prediction step that will afterwards disappear during the subsequent update step. This drastically reduces the required number of particles, making the approach numerically feasible in virtually any application. In deriving our method, we exploit the principle underlying a Bayesian tracking method called maximum likelihood Kalman filtering (MLKF) [24]. We then call the proposed method the maximum likelihood particle filter (MLPF).

The authors used the MLKF in [25] to propose a dynamic localization method which only applies to the case in which the motion model is linear and Gaussian. In this sense, the method proposed in this work can be thought of as the generalization of the method we proposed in [25] to the case of non-linear motion models. This kind of models are typically used in robotics [26], and as we show with experiments using real data, leads to great accuracy improvements.

The rest of the paper is organized as follows: In Section 2, we state the dynamic localization problem. In Section 3, we describe the particle filtering approach for solving it. In Section 4, we derive the proposed MLPF method. In Section 5, we present experimental evidence, showing the numerical advantages offered by the proposed method. Concluding remarks are given in Section 6. For ease of readability, all proofs appear in Appendix C.

## 2. Problem Description

**Figure 1:**
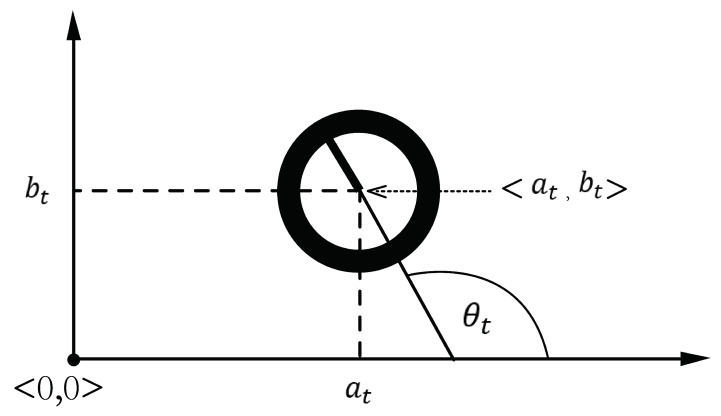
Target’s Cartesian coordinates.

We assume there is a target moving in an indoor environment. The target’s Cartesian coordinates at time *t* are $y_{t}={\left[a_{t},b_{t}\right]}^{⊤}\in R^{2}$ and $\theta_{t}\in \left(-\pi ,\pi \right]$ is its orientation in radians, as shown in Figure 1. The target’s motion model is described by the following non-linear state-transition equation

(1) $$\begin{array}{cc} y_{t+1} & =g\left(y_{t},\theta_{t},u_{t},e_{t}\right), \end{array}$$

(2) $$\begin{array}{cc} \theta_{t+1} & =h\left(y_{t},u_{t},e_{t}\right)+a\left(y_{t},u_{t},e_{t}\right)\theta_{t}+b\left(y_{t},u_{t},e_{t}\right)n_{t}, \end{array}$$

where $u_{t}$ is the target’s control input and $e_{t}={\left[{\tilde{a}}_{t},{\tilde{b}}_{t}\right]}^{⊤}$ and $n_{t}$ are process noises accounting for the model’s inaccuracy. To write the above in compact form we define the target’s pose $x_{t}={\left[y_{t}^{⊤},\theta_{t}\right]}^{⊤}\in R^{3}$ and the process noise $w_{t}=\left[e_{t}^{⊤},n_{t}\right]$. We then write

(3) $$x_{t+1}=f\left(x_{t},u_{t},w_{t}\right),$$

with *f* defined according to (1) and (2). We assume that $x_{1}=0$ and the sequence $w_{t}∼N\left(0,Q\left(u_{t}\right)\right)$ is i.i.d., with Q(u) being a diagonal positive definite matrix for each *u*.

**Remark** **1.** *We state the motion model in the general form (1) in order to make our presentation valid for the different models used in the literature. In Appendix A we briefly describe the most popular models falling into this general form.*

At each time t∈N, we obtain the following measurement

(4) $$z_{t}=m\left(y_{t}\right)+v_{t}.$$

where the map $m:R^{2}\to R^{N}$ is known and the measurement noise $v_{t}∼N\left(0,R\right)$ is i.i.d. and independent of $w_{t}$.

**Problem** **1.** *Let $Z_{T}^{⊤}=\left[z_{1}^{⊤},\cdots ,z_{T}^{⊤}\right]$ denote the set of all measurements available up to time T. The dynamic localization problem consists of using, at time T, the measurements $Z_{T}$ to obtain an estimate ${\hat{x}}_{T}$ of $x_{T}$.*

## 3. Available Solution Using Particle Filtering

As described in Section 1, the dynamic localization problem can be solved using the well-known particle filtering method [27]. In this section, we describe this approach.

The Bayesian filtering equations are initialized by $p\left(x_{1}|Z_{1}\right)=\delta \left(x_{1}\right)$, and proceed as follows

(5) $$\begin{array}{cc} p\left(x_{t}|Z_{t-1}\right) & =\int p\left(x_{t}|x_{t-1}\right)p\left(x_{t-1}|Z_{t-1}\right)dx_{t}, \end{array}$$

(6) $$\begin{array}{cc} p\left(x_{t}|Z_{t}\right) & =\frac{p\left(z_{t}|x_{t}\right)}{p\left(z_{t}|Z_{t-1}\right)}p\left(x_{t}|Z_{t-1}\right). \end{array}$$

Equations (5) and (6) are called the prediction and update steps, respectively. These equations do not have close form expressions, in general. Hence, a numeric method is needed to approximately compute them. This can be done using particle filtering. More precisely, we start with

(7) $$\begin{array}{cc} p\left(x_{1}|Z_{1};m\right) & ≃\sum_{i=1}^{I}\varpi_{1|1}^{i}\delta \left(x_{1}-x_{1|1}^{i}\right),\\ x_{1|1}^{i} & =0,\\ \varpi_{1|1}^{i} & =\frac{1}{I}. \end{array}$$

Then, at time t>1, the prediction step is computed by

(8) $$p\left(x_{t}|Z_{t-1};m\right)≃\frac{1}{I}\sum_{i=1}^{I}\delta \left(x_{t}-x_{t|t-1}^{i}\right),$$

with $x_{t|t-1}^{i}$ obtained by drawing it from the following distribution

$$x_{t|t-1}^{i}∼\sum_{i=1}^{I}\varpi_{t-1|t-1}^{i}p\left(x_{t}|x_{t-1|t-1}^{i}\right)\delta \left(x_{t}-x_{t-1|t-1}^{i}\right).$$

Also, the update step is computed by

(9) $$\begin{array}{cc} p\left(x_{t}|Z_{t};m\right) & ≃\sum_{i=1}^{I}\varpi_{t|t}^{i}\delta \left(x_{t}-x_{t|t}^{i}\right),\\ x_{t|t}^{i} & =x_{t|t-1}^{i},\\ \varpi_{t|t}^{i} & \propto p\left(y_{t}|x_{t}^{i}\right). \end{array}$$

with $\varpi_{t|t}^{i}$ normalized so that $\sum_{i=1}^{I}\varpi_{t|t}^{i}=1$.

The use of particle filtering for solving the localization problem permits accommodating the accuracy by increasing the number of particles. As mentioned in Section 1, a problem that often occurs is that the desired accuracy requires a very large number of particles. This can result in a numerically very expensive algorithm. To address this issue, in the next section we propose a variant of the particle filtering algorithm, which drastically reduces the number of particles required to achieve a given accuracy.

## 4. Proposed Algorithm

In the particle filtering algorithm described in Section 3, at time *t*, we use the approximation (9) of the updated distribution $p\left(x_{t-1}|Z_{t-1}\right)$, together with the state-transition Equation (1), to generate the approximation (8) of the predicted distribution $p\left(x_{t}|Z_{t-1}\right)$. The latter is then used in combination with the likelihood function $p\left(z_{t}|x_{t}\right)$ to approximate the new updated distribution $p\left(x_{t}|Z_{t}\right)$. This is done by weighting the *i*-th particle $x_{t|t}^{i}$ by a weight $\varpi_{t|t}^{i}$ proportional to the particle’s likelihood $p\left(y_{t}|x_{t}^{i}\right)$. As mentioned in Section 1, a drawback of this approach is that this weighting wastes many particles. In order to avoid this, we combine the prediction and update steps into a single one. More precisely, particles for approximating the updated distribution $p\left(x_{t}|Z_{t}\right)$ are directly drawn using the knowledge of the particle approximation of the previous updated distribution $p\left(x_{t-1}|Z_{t-1}\right)$, without drawing particles to represent the predicted distribution $p\left(x_{t}|Z_{t-1}\right)$. We describe below how this is done.

**Notation** **1.** *In order to simplify the notation we remove the dependence of Q on $u_{t}$, i.e., we use Q instead of $Q\left(u_{t}\right)$.*

**Notation** **2.** *Let*

$$\begin{array}{cc} f_{t-1}^{i}\left(w\right) & =f\left(x_{t-1|t-1}^{i},u_{t-1},w\right),\\ g_{t-1}^{i}\left(e\right) & =g\left(y_{t-1|t-1}^{i},\theta_{t-1|t-1}^{i},u_{t-1},e\right), \end{array}$$

*and, for ξ=h,a,b,*

$$\xi_{t-1}^{i}\left(e\right)=\xi \left(y_{t-1|t-1}^{i},u_{t-1},e\right).$$

We assume that at time *t* we know the following approximation

(10) $$p\left(x_{t-1}|Z_{t-1}\right)≃\sum_{i=1}^{I}\varpi_{t-1|t-1}^{i}\delta \left(x_{t-1}-x_{t-1|t-1}^{i}\right).$$

Our goal is to use the above to build an approximation of the form

(11) $$p\left(x_{t}|Z_{t}\right)≃\sum_{i=1}^{I}\varpi_{t|t}^{i}\delta \left(x_{t}-x_{t|t}^{i}\right).$$

Our first step is to derive a convenient decomposition of $p\left(x_{t}|Z_{t}\right)$. This is done in the following lemma.

**Lemma** **1.** *The following equality holds in the system given by (1), (2) and (4)*

(12) $$p\left(x_{t}|Z_{t}\right)=p\left(\theta_{t}|y_{t},Z_{t}\right)p\left(y_{t}|Z_{t}\right),$$

*where*

(13) $$\begin{array}{cc} p\left(y_{t}|Z_{t}\right) & \propto p\left(z_{t}|y_{t}\right)p\left(y_{t}|Z_{t-1}\right), \end{array}$$

(14) $$\begin{array}{cc} p\left(\theta_{t}|y_{t},Z_{t}\right) & \propto p\left(\theta_{t}|y_{t},Z_{t-1}\right). \end{array}$$

It follows from Lemma 1 that, in order to find an expression of $p\left(x_{t}|Z_{t}\right)$ we need expressions of $p\left(y_{t}|Z_{t-1}\right)$ and $p\left(\theta_{t}|y_{t},Z_{t}\right)$. Under the approximation (10), the desired expressions are given in the following lemma.

**Lemma** **2.** *If (10) holds, then*

(15) $$\begin{array}{cc} p\left(y_{t}|Z_{t-1}\right) & \tilde{\propto }\sum_{i=1}^{I}\psi_{t}^{i}\left(y_{t}\right), \end{array}$$

(16) $$\begin{array}{cc} p\left(\theta_{t}|y_{t},Z_{t-1}\right) & \tilde{\propto }\sum_{i=1}^{I}\psi_{t}^{i}\left(y_{t}\right)N\left(\theta_{t};\mu_{t}^{i}\left(y_{t}\right),\rho_{t}^{i}\left(y_{t}\right)\right). \end{array}$$

*where to simplify the notation we use $g_{t-1}^{-i}={\left(g_{t-1}^{i}\right)}^{-1}$, and $\tilde{\propto }$ denotes the approximately proportional sign. Also*

$$\psi_{t}^{i}\left(y_{t}\right)=\varpi_{t-1|t-1}^{i}\gamma_{t}^{i}\left(y_{t}\right)\left|\frac{\det Jg_{t-1}^{-i}\left(y_{t}\right)}{b_{t-1}^{i}\circ g_{t-1}^{-i}\left(y_{t}\right)}\right|,$$

*where J denoted the Jacobian operator,*

$$\begin{array}{cc} \rho_{t}^{i}\left(y_{t}\right) & =\frac{1}{\varsigma_{t}^{i}{\left(y_{t}\right)}^{⊤}Q^{-1}\varsigma_{t}^{i}\left(y_{t}\right)},\\ \mu_{t}^{i}\left(y_{t}\right) & =-\nu_{t}^{i}{\left(y_{t}\right)}^{⊤}Q^{-1}\varsigma_{t}^{i}\left(y_{t}\right)\rho_{t}^{i}\left(y_{t}\right)\\ \gamma_{t}^{i}\left(y_{t}\right) & =\sqrt{\frac{2\pi \rho_{t}^{i}\left(y_{t}\right)}{\det\left(2\pi Q\right)}}\exp\left(-\frac{1}{2}\nu_{t}^{i}{\left(y_{t}\right)}^{⊤}Q^{-1}\zeta_{t}^{i}\left(y_{t}\right)\right),\\ \zeta_{t}^{i}\left(y_{t}\right) & =\nu_{t}^{i}\left(y_{t}\right)+\mu_{t}^{i}\left(y_{t}\right)\varsigma_{t}^{i}\left(y_{t}\right), \end{array}$$

*and*

$$\begin{array}{cc} \nu_{t}^{i}\left(y_{t}\right) & =\left[\begin{array}{c} g_{t-1}^{-i}\left(y_{t}\right)\\ \frac{-h_{t-1}^{i}\circ g_{t-1}^{-i}\left(y_{t}\right)-a_{t-1}^{i}\circ g_{t-1}^{-i}\left(y_{t}\right)\theta_{t-1}}{b_{t-1}^{i}\circ g_{t-1}^{-i}\left(y_{t}\right)} \end{array}\right],\\ \varsigma_{t}^{i}\left(y_{t}\right) & =\left[\begin{array}{c} 0\\ 0\\ \frac{1}{b_{t-1}^{i}\circ g_{t-1}^{-i}\left(y_{t}\right)} \end{array}\right]. \end{array}$$

In order to build an approximation of the form (11) we need a method for drawing samples from $p\left(x_{t}|Z_{t}\right)$. In view of (12), this can be done by drawing samples from $p\left(\theta_{t}|y_{t},Z_{t}\right)$ and $p\left(y_{t}|Z_{t}\right)$. It follows from (16) that $p\left(\theta_{t}|y_{t},Z_{t}\right)$ is a Gaussian mixture distribution. Hence, we can readily draw samples from it. On the other hand, from (13) and (15) we obtain

$$p\left(y_{t}|Z_{t}\right)\propto p\left(z_{t}|y_{t}\right)\sum_{i=1}^{I}\psi_{t}^{i}\left(y_{t}\right),$$

from where it is not clear how to draw samples. Our strategy to go around this consists finding a convenient Gaussian approximation ${\tilde{p}}_{t}\left(y_{t}\right)$ of $p\left(y_{t}|Z_{t}\right)$, from where we can draw samples, and then weight these samples to account for the difference between ${\tilde{p}}_{t}\left(y_{t}\right)$ and $p\left(y_{t}|Z_{t}\right)$.

To derive the Gaussian approximation ${\tilde{p}}_{t}\left(y_{t}\right)$ we use (13) and proceed in three steps. In Lemma 3 we do a Gaussian approximation of $p\left(y_{t}|Z_{t-1}\right)$, in Lemma 4 we do the same with $p\left(z_{t}|y_{t}\right)$, and in Lemma 5 we combine these two approximations to obtain ${\tilde{p}}_{t}\left(y_{t}\right)$.

**Lemma** **3.** *If (10) holds, then*

$$\begin{array}{c} p\left(y_{t}|Z_{t-1}\right)≃\sum_{i=1}^{I}\varpi_{t-1|t-1}^{i}N\left(y_{t};g_{t-1}^{i}\left(0\right),Jg_{t-1}^{i}\left(0\right)Q_{e}Jg_{t-1}^{i⊤}\left(0\right)\right), \end{array}$$

*where $Q_{e}=SQS^{⊤}$ with $S=\left[I_{2},0\right]$ and $I_{2}$ denoting the 2×2 identity matrix.*

**Lemma** **4.** *The following approximation holds*

(17) $$p\left(z_{t}|y_{t}\right)\tilde{\propto }N\left(y_{t};\lambda_{t},\Lambda_{t}\right),$$

*where*

(18) $$\begin{array}{cc} \lambda_{t} & =\underset{y\in R^{2}}{\arg\max}\Xi_{t}\left(y\right), \end{array}$$

(19) $$\begin{array}{cc} \Lambda_{t} & =-{\left[\nabla^{2}\Xi_{t}\left(\lambda_{t}\right)\right]}^{-1}. \end{array}$$

*with*

$$\Xi_{t}\left(y\right)=\log N\left(z_{t};m\left(y\right),R\right).$$

Combining Lemmas 3 and 4 we obtain the Gaussian approximation of $p\left(y_{t}|Z_{t}\right)$ given in the following lemma.

**Lemma** **5.** *The following approximation holds*

$$p\left(y_{t}|Z_{t}\right)≃{\tilde{p}}_{t}\left(y_{t}\right),$$

*where*

$${\tilde{p}}_{t}\left(y_{t}\right)=\sum_{i=1}^{I}\varpi_{t-1|t-1}^{i}\alpha_{t}^{i}N\left(y_{t};\xi_{t}^{i},\Xi_{t}^{i}\right),$$

*with*

$$\begin{array}{cc} \alpha_{t}^{i} & =N\left(0;g_{t-1}^{i}\left(0\right)-\lambda_{t},\Lambda_{t}+Jg_{t-1}^{i}\left(0\right)Q_{e}Jg_{t-1}^{i⊤}\left(0\right)\right),\\ \Xi_{t}^{i} & ={\left[\Lambda_{t}^{-1}+{\left(Jg_{t-1}^{i}\left(0\right)Q_{e}Jg_{t-1}^{i⊤}\left(0\right)\right)}^{-1}\right]}^{-1},\\ \xi_{t}^{i} & =\Xi_{t}^{i}\left[\Lambda_{t}^{-1}\lambda_{t}+{\left(Jg_{t-1}^{i}\left(0\right)Q_{e}Jg_{t-1}^{i⊤}\left(0\right)\right)}^{-1}g_{t-1}^{i}\left(0\right)\right]. \end{array}$$

Combining the results in Lemmas 1, 2 and 5 we can build the desired approximation (11) following the procedure described in Algorithm 1.
**Algorithm 1** Proposed localization algorithm.At t=1, initialize $p\left(x_{1}|Z_{1}\right)$ using (7). At each t>1:1.Compute $\lambda_{t}$ and $\Lambda_{t}$ using (18) and (19).2.For each j=1,⋯,I, do:
(a)Pick an index $i\in \left\{1,\cdots ,I\right\}$ by drawing it from the discrete distribution such that *i* has probability proportional to $\varpi_{t-1|t-1}^{i}\alpha_{t}^{i}$.(b)Draw $y_{t|t}^{j}$ from $N\left(y_{t};\xi_{t}^{i},\Xi_{t}^{i}\right)$.(c)Compute the particle weight using $\varpi_{t|t}^{j}=\frac{p\left(y_{t|t}^{j}|Z_{t}\right)}{{\tilde{p}}_{t}\left(y_{t|t}^{j}\right)}$.(d)Pick an index $i\in \left\{1,\cdots ,I\right\}$ by drawing it from the discrete distribution such that *i* has probability proportional to $\psi_{t}^{i}\left(y_{t|t}^{j}\right)$.(e)Draw $\theta_{t|t}^{j}$ from $N\left(\mu_{t}^{i}\left(y_{t|t}^{j}\right),\rho_{t}^{i}\left(y_{t|t}^{j}\right)\right)$.(f)Put $x_{t|t}^{j}={\left[y_{t|t}^{j⊤},\theta_{t|t}^{j}\right]}^{⊤}$.3.Normalize the weights so that $\sum_{j=1}^{I}\varpi_{t|t}^{j}=1$.4.Estimate the position using ${\hat{x}}_{t}=\sum_{i=1}^{I}\varpi_{t|t}^{i}x_{t|t}^{i}$.

## 5. Experimental Validation

In this section, we evaluate the performance of our proposed indoor localization method. To this end, we compare it with that of two methods based on particle filtering (PF). The first one is a newly proposed RSS-based method described in [20], which we refer to as PF-RSS. In order to assess the advantage resulting from using CSI measurements instead of RSS ones, the second method is the CSI-based version the PF-RSS one. It is obtained by using the PF method, described in Section 3, instead of the proposed MLPF method. We refer to it as PF-CSI. We also consider the dynamic localization method from [25], which we call MLKF, and only applies to linear Gaussian motion models like the one given in Appendix B. We also consider the localization performance obtained via static localization. To this end, we use the static localization method proposed in [25], which, as shown in that work, outperforms other available static positioning methods like FILA [28], DeepFi [29], and PhaseFi [30].

As APs we used TP-Link TL-WDR4310 routers with the OpenWrt platform installed. To acquire CSI values we use the Atheros CSI tool [31]. It provides CSI values of 56 subcarriers, and for each one, two 10 bit values are used to represent its phase and amplitude. Motion capture cameras with millimeter-level accuracy are used to obtain the ground truth position.

For the target we use a two-wheel robot controlled by a NVIDIA Jetson Nano developer board, equipped with an Atheros AR9590 network interface controller. We consider a target whose motion is described by the velocity model given in Appendix A.1, with $\sigma_{v}^{2}=0.36$, $\sigma_{\omega }^{2}=0.072$ and $\sigma_{\gamma }^{2}=0.072$. To generate the measurements we pass the acquired CSI phase values through the linear calibration stage proposed in [30] (*§* II.B), then unwrap the resulting phases and use the resulting phase differences as fingerprints. The whole procedure for generating fingerprints is described in [25]. The measurement model is given by the following Gaussian kernel expansion

(20) $$m\left(y_{t}\right)=\sum_{n=1}^{N}\kappa_{n}\exp\left(-\chi {∥y_{t}-p_{n}∥}^{2}\right),$$

where $p_{n}\in R^{2}$, n∈[1,2,⋯,N], denote the positions used to build the fingerprint database. Following [32] (Chapter 4.3.2) we choose $\chi =\frac{1}{2}I^{1/3}$. Also,$\kappa ={\left[\kappa_{1},\cdots ,\kappa_{N}\right]}^{⊤}$ is computed so that, for each n=1,⋯,N, $m\left(p_{n}\right)$ matches the CSI measured at $p_{n}$.

As performance metric we use the mean squared localization error ϵ, i.e., if the estimated pose at time *t* is ${\hat{x}}_{t}={\left[{\hat{y}}_{t}^{⊤},{\hat{\theta }}_{t}\right]}^{⊤}$ and the ground truth is $y_{t}$, then

$$\epsilon =\frac{1}{T}\sum_{t=1}^{T}{∥{\hat{y}}_{t}-y_{t}∥}^{2}.$$

For the PF method, following [23] we use I=1000 particles. Also, for the MLKF method, we use the linear Gaussian motion model described in Appendix B, with $\sigma_{p}^{2}=0.12$ and $\sigma_{\iota }^{2}=0.25$.

**Figure 2:**
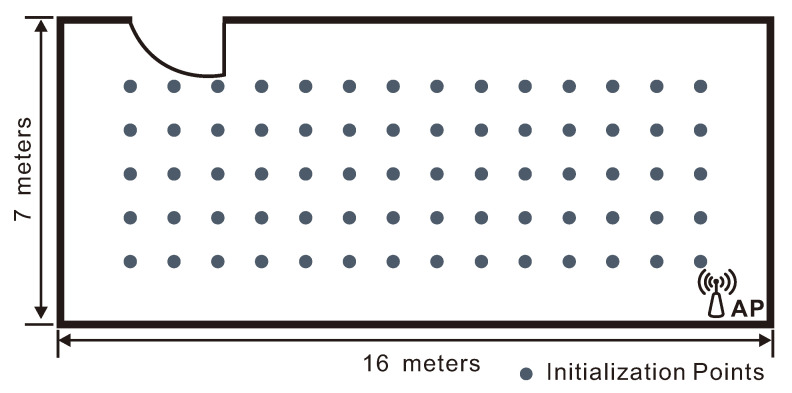
Layout and database points of the empty room. The access point (AP) is placed at the lower-right corner.

In the first experiment, we aim to evaluate the performance in large environments. To this end we use an empty room of 7×16 m${}^{2}$. Only one AP is placed at the corner of the room as shown in Figure 2. To build the fingerprinting database, we measured CSI’s at N=70 positions, one meter apart from each other, on a grid as also shown in the same figure. As the target moves within the room, we estimate its position every τ=0.5 seconds.

**Figure 3:**
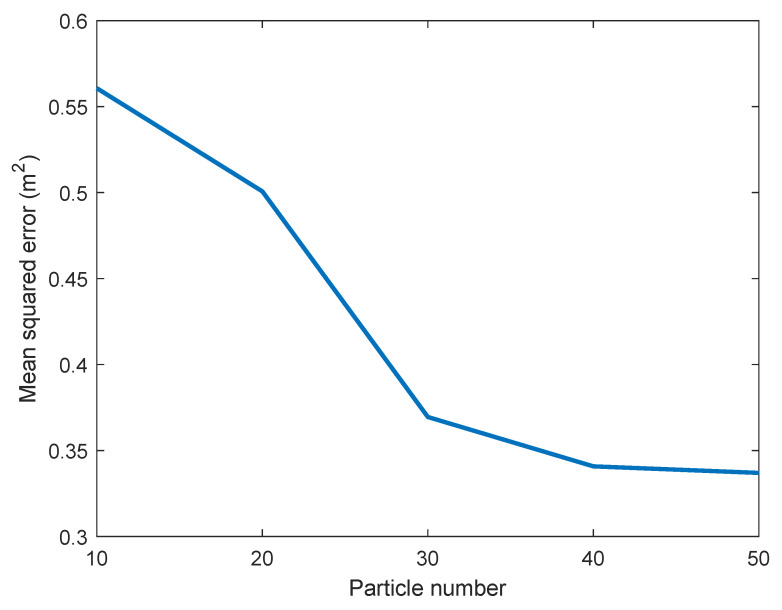
Impact of particle numbers on positioning accuracy.

**Figure 4:**
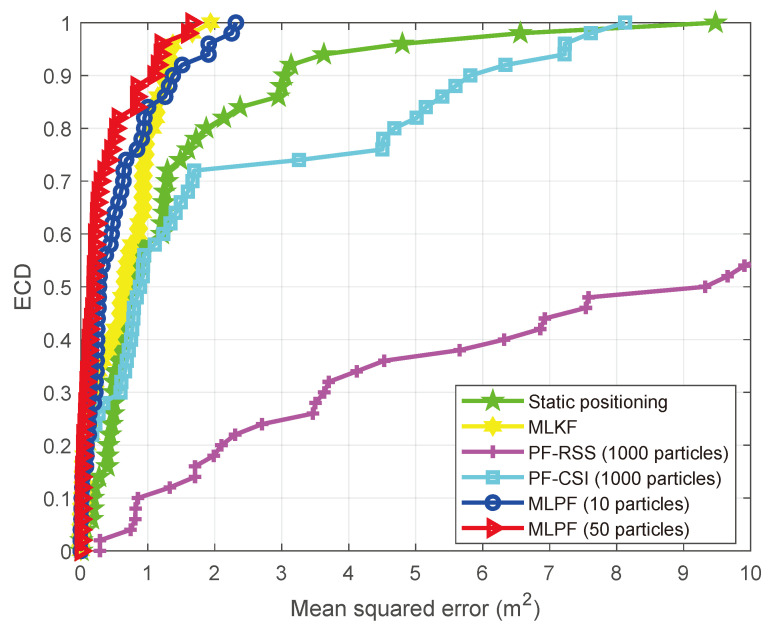
Positioning error cumulative distribution (ECD) (empty room).

**Figure 5:**
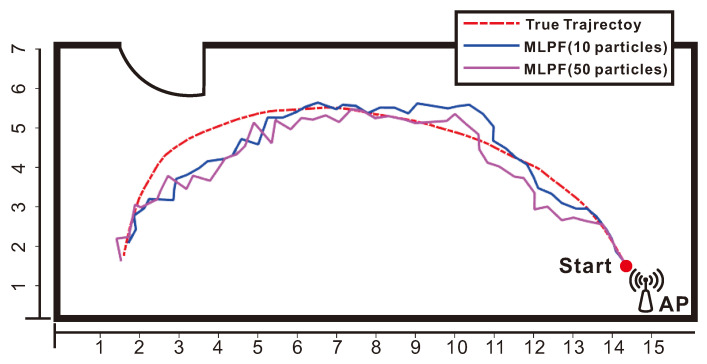
Tracking result of maximum likelihood particle filter (MLPF) with 10 and 50 particles in an empty room.

As we can see from Table 1, the proposed MLPF method, with only 10 particles, clearly outperforms the outcomes of static positioning and particle filtering. Its performance is comparable to that of the MLKF. However, it outperforms the latter if we increase the number of particles to 50. This is due to the use of a non-linear motion model which yields more accurate statistical knowledge of the target’s position at each sample time. Also, although particle filtering should yield the theoretically optimal result if the number of particles is large enough, the very large number of required particles result in that, even with 1000 particles, its accuracy is significantly inferior than that of the proposed MLPF method with 10 particles. Figure 3 shows how the positioning accuracy of the proposed method improves with the number of particles. In Figure 4 we show the error cumulative distribution (ECD) of each method, i.e., for each error (in meters), we show the proportion of positions whose localization error is smaller than that error. The estimated trajectory and ground truth of the experiment are shown in Figure 5.

**Figure 6:**
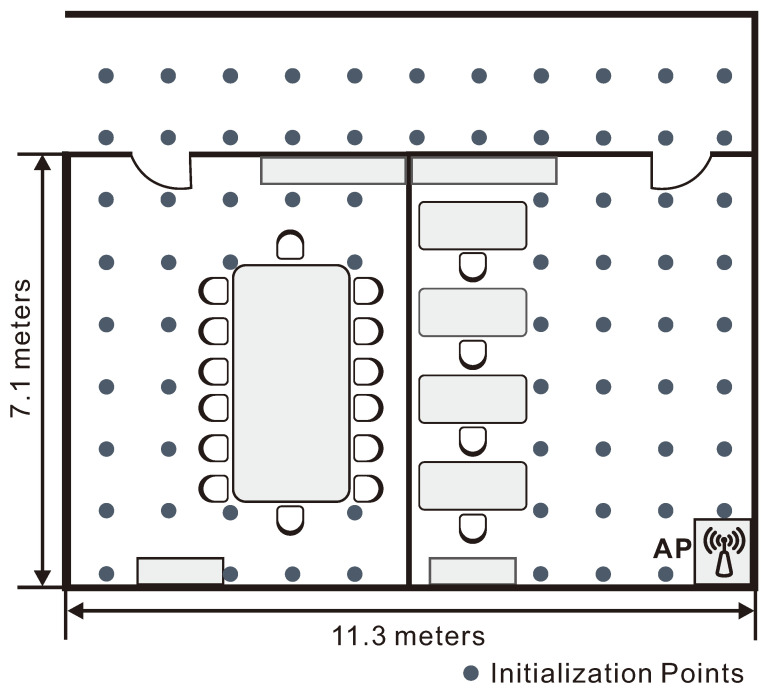
Layout and database points for the two office rooms. The AP is placed at the lower-right corner of the right room.

The second experiment aims to show the performance of our method in living and working environments. To this end we use two adjacent offices of 7.1×11.3 m${}^{2}$ connected by a corridor. We build the fingerprint database using N=72 positions, as shown in Figure 6, together with the AP’s location.

**Figure 7:**
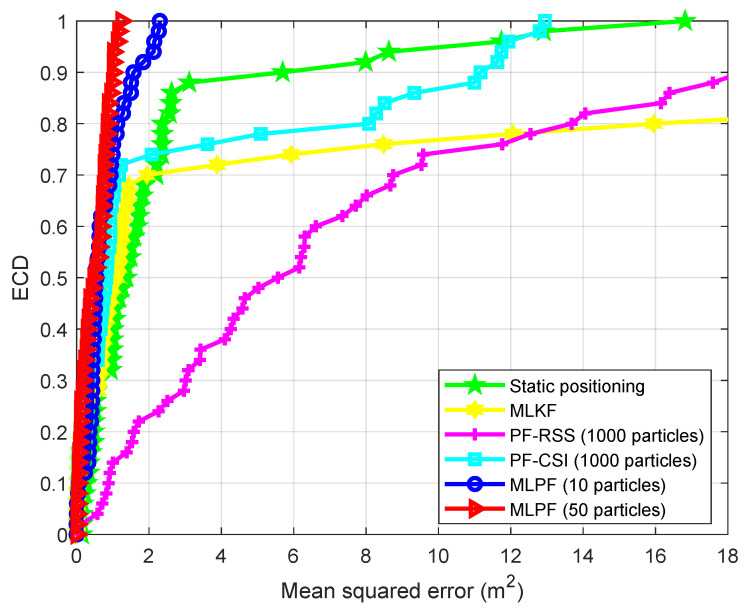
Positioning ECD (office rooms).

**Figure 8:**
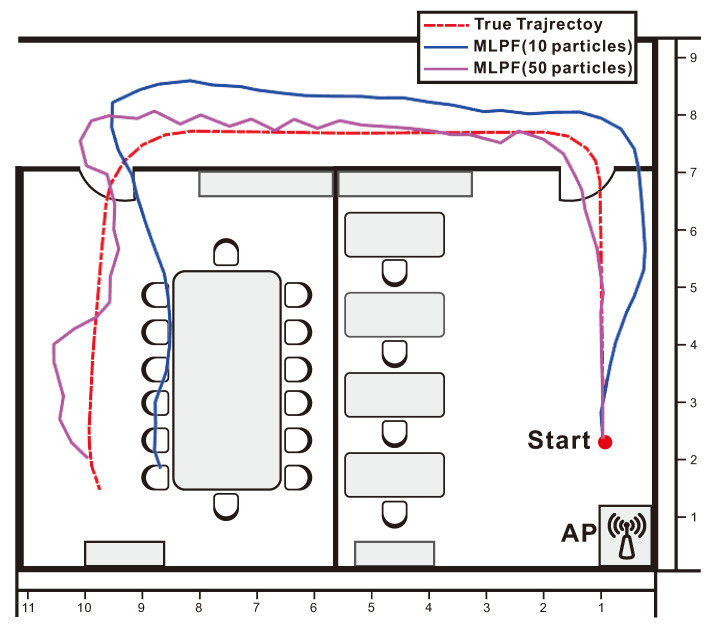
Tracking result of MLPF with 10 and 50 particles in the office rooms.

We see from Table 2 that, while in the empty room the proposed method with 10 particles performed similarly to the MLKF method, in this second experiment it largely outperforms the latter. The table also shows the clear advantage of the proposed method over its rivals. The reason for this advantage is as follows. Due to the signal distortion and attenuation caused by multipaths, walls and furnitures, the localization information provided by measurements is not enough to yield accurate estimates at certain locations. This can be seen in the ECD shown in Figure 7. At those locations, dynamic localization methods resort to motion model information to produce an estimate. Figure 7 shows that only the proposed MLPF method can produce accurate estimates at those locations. This is because it is the only method that is able to make full use of the rich information provided by a non-linear motion model. While methods based on PF potentially have the same possibility, the extremely large number of required particles make this unfeasible in practice. To conclude the experiment, we show in Figure 8 the ground truth and estimated trajectories.

## 6. Conclusions

We proposed a new indoor positioning method, which we called maximum likelihood particle filtering. Its essential idea consists of combing the prediction and updates steps of a traditional particle filter into a single step, which requires solving a maximum likelihood estimation problem. This results in a better utilization of particles. The method so obtained achieves high accuracy indoor positioning with a drastically smaller number of particles in comparison with particle filtering. This makes it numerically feasible for real-time applications. We validated our claims in a WiFi indoor positioning experiment using real data. Our experiment show that our method leads to great accuracy improvements achieving sub-meter level indoor localization accuracy, which exceeds the requirements of the 5G NR Release 16 standard from 3GPP [33].

## Figures and Tables

**Table 1:** Positioning Error (empty room).

Methods	Mean Squared Error [m${}^{2}$]	90% Acc. [m${}^{2}$]
Static positioning	1.4153	3.0437
MLKF	0.6506	1.2848
PF-RSS (1000 particles)	12.1310	28.3569
PF-CSI (1000 particles)	2.0834	5.8151
MLPF (10 particles)	0.5607	1.3724
MLPF (50 particles)	0.3370	1.0891

**Table 2:** Positioning Error (office rooms).

Methods	Mean Squared Error [m${}^{2}$]	90% Acc. [m${}^{2}$]
Static positioning	2.3962	5.6935
MLKF	11.7255	50.8279
PF-RSS (1000 particles)	8.2366	18.3872
PF-CSI (1000 particles)	2.9418	11.1606
MLPF (10 particles)	0.7755	1.5712
MLPF (50 particles)	0.4939	0.9970

## Data Availability

Not applicable.

## Appendix A. Non-Linear Robot Motion Models

### Appendix A.1. Velocity Model

The input is $u_{t}=\left[v_{t},\omega_{t}\right]$, with $v_{t}$ and $\omega_{t}$ being the robot’s measured linear and angular speeds, respectively. Also $e_{t}=\left[{\tilde{v}}_{t},{\tilde{\omega }}_{t}\right]$, $n_{t}={\tilde{\gamma }}_{t}$, $Q\left(u_{t}\right)=\mathrm{diag}\left(\sigma_{v}^{2},\sigma_{\omega }^{2},\sigma_{\gamma }^{2}\right)$ with

$$\begin{array}{cc} \sigma_{v}^{2} & =\alpha_{1}\left|v_{t}\right|+\alpha_{2}\left|\omega_{t}\right|,\\ \sigma_{\omega }^{2} & =\alpha_{3}\left|v_{t}\right|+\alpha_{4}\left|\omega_{t}\right|,\\ \sigma_{\gamma }^{2} & =\alpha_{5}\left|v_{t}\right|+\alpha_{6}\left|\omega_{t}\right|, \end{array}$$

and $\alpha_{1},\cdots ,\alpha_{6}$ being system parameters. The motion model is given by

$$\begin{array}{cc} a_{t+1} & =a_{t}-\frac{{\hat{v}}_{t}}{{\hat{\omega }}_{t}}\sin\theta_{t}+\frac{{\hat{v}}_{t}}{{\hat{\omega }}_{t}}\sin\left(\theta_{t}+{\hat{\omega }}_{t}\tau \right),\\ b_{t+1} & =b_{t}+\frac{{\hat{v}}_{t}}{{\hat{\omega }}_{t}}\cos\theta_{t}-\frac{{\hat{v}}_{t}}{{\hat{\omega }}_{t}}\cos\left(\theta_{t}+{\hat{\omega }}_{t}\tau \right),\\ \theta_{t+1} & =\theta_{t}+\left({\hat{\omega }}_{t}+{\tilde{\gamma }}_{t}\right)\tau , \end{array}$$

where τ denotes the sampling time and

$$\begin{array}{cc} {\hat{v}}_{t} & =v_{t}+{\tilde{v}}_{t},\\ {\hat{\omega }}_{t} & =\omega_{t}+{\tilde{\omega }}_{t}. \end{array}$$

### Appendix A.2. Odometry Model

In this case $u_{t}={\left[{\bar{x}}_{t+1}^{⊤},{\bar{x}}_{t}^{⊤}\right]}^{⊤}$, where ${\bar{x}}_{t}={\left[{\bar{a}}_{t},{\bar{b}}_{t},{\bar{\theta }}_{t}\right]}^{⊤}$ denotes the odometry measurement at time *t*. Using $u_{t}$ we compute

$$\begin{array}{cc} \delta_{\mathrm{rot}1} & =\arctan\frac{{\bar{b}}_{t+1}-{\bar{b}}_{t}}{{\bar{a}}_{t+1}-{\bar{a}}_{t}}-{\bar{\theta }}_{t},\\ \delta_{\mathrm{trans}} & =\sqrt{{\left({\bar{a}}_{t+1}-{\bar{a}}_{t}\right)}^{2}+{\left({\bar{b}}_{t+1}-{\bar{b}}_{t}\right)}^{2}},\\ \delta_{\mathrm{rot}1} & ={\bar{\theta }}_{t+1}-{\bar{\theta }}_{t}-\delta_{\mathrm{rot}1}. \end{array}$$

Then, $w_{t}=\left[{\tilde{\delta }}_{\mathrm{rot}1},{\tilde{\delta }}_{\mathrm{trans}},{\tilde{\delta }}_{\mathrm{rot}2}\right]$ and $Q\left(u_{t}\right)=\mathrm{diag}\left(\sigma_{\mathrm{rot}1}^{2},\sigma_{\mathrm{trans}}^{2},\sigma_{\mathrm{rot}2}^{2}\right)$ with

$$\begin{array}{cc} \sigma_{\mathrm{rot}1}^{2} & =\alpha_{1}\left|{\hat{\delta }}_{\mathrm{rot}1}\right|+\alpha_{2}\left|{\hat{\delta }}_{\mathrm{trans}}\right|,\\ \sigma_{\mathrm{trans}}^{2} & =\alpha_{3}\left|{\hat{\delta }}_{\mathrm{trans}}\right|+\alpha_{4}\left|{\hat{\delta }}_{\mathrm{rot}1}+{\hat{\delta }}_{\mathrm{rot}2}\right|,\\ \sigma_{\mathrm{rot}2}^{2} & =\alpha_{5}\left|{\hat{\delta }}_{\mathrm{rot}2}\right|+\alpha_{6}\left|{\hat{\delta }}_{\mathrm{trans}}\right|, \end{array}$$

and $\alpha_{1},\cdots ,\alpha_{6}$ being system parameters. The motion model is given by

$$\begin{array}{cc} a_{t+1} & =a_{t}+{\hat{\delta }}_{\mathrm{trans}}\cos\left(\theta_{t}+{\hat{\delta }}_{\mathrm{rot}1}\right),\\ b_{t+1} & =b_{t}+{\hat{\delta }}_{\mathrm{trans}}\sin\left(\theta_{t}+{\hat{\delta }}_{\mathrm{rot}1}\right),\\ \theta_{t+1} & =\theta_{t}+{\hat{\delta }}_{\mathrm{rot}1}+{\hat{\delta }}_{\mathrm{rot}2}, \end{array}$$

where

$$\begin{array}{cc} {\hat{\delta }}_{\mathrm{rot}1} & =\delta_{\mathrm{rot}1}+{\tilde{\delta }}_{\mathrm{rot}1},\\ {\hat{\delta }}_{\mathrm{trans}} & =\delta_{\mathrm{trans}}+{\tilde{\delta }}_{\mathrm{trans}},\\ \delta_{\mathrm{rot}2} & =\delta_{\mathrm{rot}2}+{\tilde{\delta }}_{\mathrm{rot}2}. \end{array}$$

### Appendix A.3. Differential Drive Model

As in the velocity model, $u_{t}=\left[v_{t},\omega_{t}\right]$. Also $w_{t}=\left[{\tilde{v}}_{t},{\tilde{\eta }}_{t},{\tilde{\omega }}_{t}\right]$, where $Q\left(u_{t}\right)=\mathrm{diag}\left(\sigma_{v}^{2},\sigma_{\eta }^{2},\sigma_{\omega }^{2}\right)$ with $\sigma_{v}^{2}$, $\sigma_{\eta }^{2}$, and $\sigma_{\omega }^{2}$ being system parameters. The motion model is given by

$$\begin{array}{cc} a_{t+1} & =a_{t}+\tau \cos\left(\theta_{t}\right)\left(v_{t}+{\tilde{v}}_{t}\right)-\tau \sin\left(\theta_{t}\right){\tilde{\eta }}_{t},\\ b_{t+1} & =b_{t}+\tau \sin\left(\theta_{t}\right)\left(v_{t}+{\tilde{v}}_{t}\right)+\tau \cos\left(\theta_{t}\right){\tilde{\eta }}_{t},\\ \theta_{t+1} & =\theta_{t}+\tau {\tilde{\omega }}_{t}, \end{array}$$

where τ denotes the sampling time.

## Appendix B. Linear Motion Model

In the linear motion model, $p_{\zeta }$ and $v_{\zeta }$, ζ∈{x,y}, denote the position and velocity, respectively, in the ζ axis. The model is given by

$$\begin{array}{cc} p_{x}\left(t+1\right) & =p_{x}\left(t\right)+\tau v_{x}\left(t\right)+w_{x}\left(t\right),\\ p_{y}\left(t+1\right) & =p_{y}\left(t\right)+\tau v_{y}\left(t\right)+w_{y}\left(t\right),\\ v_{x}\left(t+1\right) & =v_{x}\left(t\right)+\iota_{x}\left(t\right),\\ v_{y}\left(t+1\right) & =v_{y}\left(t\right)+\iota_{y}\left(t\right), \end{array}$$

where $w_{x}$, $w_{y}$, $\iota_{x}$ and $\iota_{y}$ are mutually independent, $w_{x}∼w_{y}∼N\left(0,\sigma_{p}^{2}\right)$, and $\iota_{x}∼\iota_{y}∼N\left(0,\sigma_{\iota }^{2}\right)$.

## Appendix C. Proofs

**Proof of Lemma 1.** We have

$$\begin{array}{cc} p\left(x_{t}|Z_{t}\right) & =p\left(\theta_{t}|y_{t},Z_{t}\right)p\left(y_{t}|Z_{t}\right). \end{array}$$

Now

$$\begin{array}{cc} p\left(y_{t}|Z_{t}\right) & =\frac{1}{p\left(z_{t}|Z_{t-1}\right)}p\left(y_{t},z_{t}|Z_{t-1}\right)\\ \; & \propto p\left(z_{t}|y_{t}\right)p\left(y_{t}|Z_{t-1}\right), \end{array}$$

and

$$\begin{array}{cc} p\left(\theta_{t}|y_{t},Z_{t}\right) & \propto p\left(\theta_{t},y_{t}|Z_{t}\right)\\ \; & \propto p\left(\theta_{t},y_{t},z_{t}|Z_{t-1}\right)\\ \; & \propto p\left(z_{t}|\theta_{t},y_{t}\right)p\left(\theta_{t},y_{t}|Z_{t-1}\right)\\ \; & \propto p\left(z_{t}|y_{t}\right)p\left(\theta_{t},y_{t}|Z_{t-1}\right)\\ \; & \propto p\left(\theta_{t},y_{t}|Z_{t-1}\right)\\ \; & \propto p\left(\theta_{t}|y_{t},Z_{t-1}\right). \end{array}$$

□

**Proof of Lemma 2.** We split the proof in steps:

1. It follows straightforwardly from [34] (§ 5.4) that
  $$\begin{array}{c} p\left(x_{t}|x_{t-1|t-1}^{i}\right)=\sum_{w:x_{t}=f_{t-1}\left(w\right)}{\left|\det Jf_{t-1}^{i}\left(w\right)\right|}^{-1}p\left(w_{t-1}=w\right). \end{array}$$
  Since the variance of $n_{t-1}$ is small, we can do the following approximation,
  (A1) $$\begin{array}{cc} p\left(x_{t}|x_{t-1|t-1}^{i}\right)≃ & {\left|\det Jf_{t-1}^{i}\left(f_{t-1}^{-i}\left(x_{t}\right)\right)\right|}^{-1}N\left(f_{t-1}^{-i}\left(x_{t}\right);0,Q\right)\\ ≃ & \left|\det Jf_{t-1}^{-i}\left(x_{t}\right)\right|N\left(f_{t-1}^{-i}\left(x_{t}\right);0,Q\right) \end{array}$$
  where to simplify the notation we used $f_{t-1}^{-i}={\left(f_{t-1}^{i}\right)}^{-1}$.
2. We have
  (A2) $$\begin{array}{cc} f_{t-1}^{-i}\left(x_{t}\right)= & \left[\begin{array}{c} g_{t-1}^{-i}\left(y_{t}\right)\\ \frac{\theta_{t}-h_{t-1}^{i}\circ g_{t-1}^{-i}\left(y_{t}\right)-a_{t-1}^{i}\circ g_{t-1}^{-i}\left(y_{t}\right)\theta_{t-1}}{b_{t-1}^{i}\circ g_{t-1}^{-i}\left(y_{t}\right)} \end{array}\right] \end{array}$$
  (A3) $$\begin{array}{cc} = & \nu_{t}^{i}\left(y_{t}\right)+\varsigma_{t}^{i}\left(y_{t}\right)\theta_{t}. \end{array}$$
  Then, from (A2),
  $$Jf_{t-1}^{-i}\left(x_{t}\right)=\left[\begin{array}{cc} Jg_{t-1}^{-i}\left(y_{t}\right) & 0\\ {}* & \frac{1}{b_{t-1}^{i}\circ g_{t-1}^{-i}\left(y_{t}\right)} \end{array}\right],$$
  where ∗ denotes a non-zero term whose value is irrelevant. Then
  (A4) $$\left|\det Jf_{t-1}^{-i}\left(x_{t}\right)\right|=\left|\frac{\det Jg_{t-1}^{-i}\left(y_{t}\right)}{b_{t-1}^{i}\circ g_{t-1}^{-i}\left(y_{t}\right)}\right|.$$
3. Now, using (10) and (A1), we get
  (A5) $$\begin{array}{cc} p\left(y_{t},\theta_{t}|Z_{t-1}\right)= & p\left(x_{t}|Z_{t-1}\right)\\ = & \int p\left(x_{t}|x_{t-1}\right)p\left(x_{t-1}|Z_{t-1}\right)dy_{t-1}\theta_{t-1}\\ ≃ & \sum_{i=1}^{I}\varpi_{t-1|t-1}^{i}p\left(x_{t}|x_{t-1|t-1}^{i}\right)\\ ≃ & \sum_{i=1}^{I}\varpi_{t-1|t-1}^{i}\left|\det Jf_{t-1}^{-i}\left(x_{t}\right)\right|N\left(f_{t-1}^{-i}\left(x_{t}\right);0,Q\right). \end{array}$$
  But, from (A3),
  $$N\left(f_{t-1}^{-i}\left(x_{t}\right);0,Q\right)=N\left(\nu_{t}^{i}\left(y_{t}\right)+\varsigma_{t}^{i}\left(y_{t}\right)\theta_{t};0,Q\right).$$
  Now
  $$\begin{array}{cc} \; & {\left(\nu_{t}^{i}\left(y_{t}\right)+\varsigma_{t}^{i}\left(y_{t}\right)\theta_{t}\right)}^{⊤}Q^{-1}\left(\nu_{t}^{i}\left(y_{t}\right)+\varsigma_{t}^{i}\left(y_{t}\right)\theta_{t}\right)\\ = & \varsigma_{t}^{i}{\left(y_{t}\right)}^{⊤}Q^{-1}\varsigma_{t}^{i}\left(y_{t}\right)\theta_{t}^{2}+2\nu_{t}^{i}{\left(y_{t}\right)}^{⊤}Q^{-1}\varsigma_{t}^{i}\left(y_{t}\right)\theta_{t}+\nu_{t}^{i}{\left(y_{t}\right)}^{⊤}Q^{-1}\nu_{t}^{i}\left(y_{t}\right)\\ = & \varsigma_{t}^{i}{\left(y_{t}\right)}^{⊤}Q^{-1}\varsigma_{t}^{i}\left(y_{t}\right)\theta_{t}^{2}-2\varsigma_{t}^{i}{\left(y_{t}\right)}^{⊤}Q^{-1}\varsigma_{t}^{i}\left(y_{t}\right)\mu_{t}^{i}\left(y_{t}\right)\theta_{t}\\ \; & +\varsigma_{t}^{i}{\left(y_{t}\right)}^{⊤}Q^{-1}\varsigma_{t}^{i}\left(y_{t}\right)\mu_{t}^{i}{\left(y_{t}\right)}^{2}+\nu_{t}^{i}{\left(y_{t}\right)}^{⊤}Q^{-1}\nu_{t}^{i}\left(y_{t}\right)-\varsigma_{t}^{i}{\left(y_{t}\right)}^{⊤}Q^{-1}\varsigma_{t}^{i}\left(y_{t}\right)\mu_{t}^{i}{\left(y_{t}\right)}^{2}\\ = & \frac{{\left(\theta_{t}-\mu_{t}^{i}\left(y_{t}\right)\right)}^{2}}{\rho_{t}^{i}\left(y_{t}\right)}+\nu_{t}^{i}{\left(y_{t}\right)}^{⊤}Q^{-1}\nu_{t}^{i}\left(y_{t}\right)+\nu_{t}^{i}{\left(y_{t}\right)}^{⊤}Q^{-1}\varsigma_{t}^{i}\left(y_{t}\right)\mu_{t}^{i}\left(y_{t}\right)\\ = & \frac{{\left(\theta_{t}-\mu_{t}^{i}\left(y_{t}\right)\right)}^{2}}{\rho_{t}^{i}\left(y_{t}\right)}+\nu_{t}^{i}{\left(y_{t}\right)}^{⊤}Q^{-1}\left(\nu_{t}^{i}\left(y_{t}\right)+\varsigma_{t}^{i}\left(y_{t}\right)\mu_{t}^{i}\left(y_{t}\right)\right) \end{array}$$
  Hence
  (A6) $$\begin{array}{cc} N\left(f_{t-1}^{-i}\left(x_{t}\right);0,Q\right)= & \frac{1}{\sqrt{\det\left(2\pi Q\right)}}\exp\left(-\frac{{\left(\theta_{t}-\mu_{t}^{i}\left(y_{t}\right)\right)}^{2}}{2\rho_{t}^{i}\left(y_{t}\right)}\right)\times \exp\left(-\frac{1}{2}\nu_{t}^{i}{\left(y_{t}\right)}^{⊤}Q^{-1}\zeta_{t}^{i}\left(y_{t}\right)\right)\\ = & \gamma_{t}^{i}\left(y_{t}\right)N\left(\theta_{t};\mu_{t}^{i}\left(y_{t}\right),\rho_{t}^{i}\left(y_{t}\right)\right), \end{array}$$
4. Putting (A4) and (A6) into (A5) we obtain
  $$p\left(y_{t},\theta_{t}|Z_{t-1}\right)\tilde{\propto }\sum_{i=1}^{I}\psi_{t}^{i}\left(y_{t}\right)N\left(\theta_{t};\mu_{t}^{i}\left(y_{t}\right),\rho_{t}^{i}\left(y_{t}^{i}\right)\right),$$
  and the result follows.

□

**Proof of Lemma 3.** If $x_{t-1}=x_{t-1|t-1}^{i}$, doing a first order Taylor expansion we obtain

$$\begin{array}{cc} y_{t} & =g_{t-1}^{i}\left(e_{t}\right)\\ \; & ≃g_{t-1}^{i}\left(0\right)+Jg_{t-1}^{i}\left(0\right)e_{t}. \end{array}$$

So

$$\begin{array}{cc} p\left(y_{t}|x_{t-1|t-1}^{i}\right) & ≃N\left(y_{t};g_{t-1}^{i}\left(0\right),Jg_{t-1}^{i}\left(0\right)Q_{e}Jg_{t-1}^{i⊤}\left(0\right)\right). \end{array}$$

The result then follows since

$$\begin{array}{cc} p\left(y_{t}|Z_{t-1}\right)= & \sum_{i=1}^{I}\varpi_{t-1|t-1}^{i}p\left(y_{t}|x_{t-1|t-1}^{i}\right)\\ = & \sum_{i=1}^{I}\varpi_{t-1|t-1}^{i}N\left(y_{t};g_{t-1}^{i}\left(0\right),Jg_{t-1}^{i}\left(0\right)Q_{e}Jg_{t-1}^{i⊤}\left(0\right)\right). \end{array}$$

□

**Proof of Lemma 4.** Let

$$\begin{array}{cc} L_{t}\left(y\right) & =p\left(z_{t}|y_{t}=y\right). \end{array}$$

Recall that *N* is the dimension of $z_{t}$. It follows from ([24], Theorem 6) that, for all $\epsilon \in R^{2}$, we can do the following approximation

(A7) $$\lim_{N\to \infty }\frac{1}{L_{t}\left(\lambda_{t}\right)}L_{t}\left({\left(\Lambda_{t}\right)}^{-1/2}\epsilon +\lambda_{t}\right)\overset{w.p.1}{=}\exp\left(-\frac{\epsilon^{T}\epsilon }{2}\right),$$

with

$$\begin{array}{cc} \Xi_{t}\left(y\right) & =\log L_{t}\left(y\right)\\ \; & =\log N\left(z_{t};m\left(y\right),R\right). \end{array}$$

In view of the above, for large *N* we have

(A8) $$L_{t}\left(y\right)≃L_{t}\left(\lambda_{t}^{i}\right)\exp\left(-\frac{1}{2}{\left(y-\lambda_{t}\right)}^{T}\Lambda_{t}^{-1}\left(y-\lambda_{t}\right)\right),$$

Hence

$$\begin{array}{cc} p\left(z_{t}|y_{t}\right) & =L_{t}\left(y_{t}\right)\\ \; & \tilde{\propto }N\left(y_{t};\lambda_{t},\Lambda_{t}\right). \end{array}$$

□

**Proof of Lemma 5.** From the above we get

$$\begin{array}{cc} p\left(y_{t}|Z_{t}\right)\propto & p\left(z_{t}|y_{t}\right)p\left(y_{t}|Z_{t-1}\right)\\ ≃ & \sum_{i=1}^{I}\varpi_{t-1|t-1}^{i}N\left(y_{t};\lambda_{t},\Lambda_{t}\right)\times N\left(y_{t};g_{t-1}^{i}\left(0\right),Jg_{t-1}^{i}\left(0\right)Q_{e}Jg_{t-1}^{i⊤}\left(0\right)\right). \end{array}$$

The result then follows since, from [35] (§ 8.1.8),

$$\begin{array}{c} N\left(y_{t};\lambda_{t},\Lambda_{t}\right)N\left(y_{t};g_{t-1}^{i}\left(0\right),Jg_{t-1}^{i}\left(0\right)Q_{e}Jg_{t-1}^{i⊤}\left(0\right)\right)=\alpha_{t}^{i}N\left(y_{t};\xi_{t}^{i},\Xi_{t}^{i}\right). \end{array}$$

□

## References

[1] Saab S.S., Nakad Z.S. (2010). A standalone RFID indoor positioning system using passive tags. IEEE Trans. Ind. Electron..

[2] Seco F., Jiménez A.R. (2018). Smartphone-Based Cooperative Indoor Localization with RFID Technology. Sensors.

[3] Hallberg J., Nilsson M., Synnes K. Positioning with bluetooth. Proceedings of the 10th International Conference on Telecommunications 2003 (ICT 2003).

[4] Yuan Z., Yang J., You L., Qi L., Naser E.S. (2016). Smartphone-Based Indoor Localization with Bluetooth Low Energy Beacons. Sensors.

[5] Gigl T., Janssen G.J., Dizdarevic V., Witrisal K., Irahhauten Z. Analysis of a UWB indoor positioning system based on received signal strength. Proceedings of the 2007 4th Workshop on Positioning, Navigation and Communication.

[6] Mazhar F., Khan M.G., Sällberg B. (2017). Precise indoor positioning using UWB: A review of methods, algorithms and implementations. Wirel. Pers. Commun..

[7] Yang C., Shao H.R. (2015). WiFi-based indoor positioning. IEEE Commun. Mag..

[8] Jian C., Gang O., Ao P., Zheng L., Shi J. (2018). An INS/WiFi Indoor Localization System Based on the Weighted Least Squares. Sensors.

[9] Yang Z., Zhou Z., Liu Y. (2013). From RSSI to CSI: Indoor localization via channel response. ACM Comput. Surv. (CSUR).

[10] Zafari F., Gkelias A., Leung K.K. (2019). A survey of indoor localization systems and technologies. IEEE Commun. Surv. Tutor..

[11] Schatzberg U., Banin L., Amizur Y. Enhanced WiFi ToF indoor positioning system with MEMS-based INS and pedometric information. Proceedings of the 2014 IEEE/ION Position, Location and Navigation Symposium-PLANS 2014.

[12] Kotaru M., Joshi K., Bharadia D., Katti S. Spotfi: Decimeter level localization using wifi. Proceedings of the 2015 ACM Conference on Special Interest Group on Data Communication.

[13] Xia S., Liu Y., Yuan G., Zhu M., Wang Z. (2017). Indoor fingerprint positioning based on Wi-Fi: An overview. ISPRS Int. J. Geo-Inf..

[14] Wen Y., Tian X., Wang X., Lu S. Fundamental limits of RSS fingerprinting based indoor localization. Proceedings of the 2015 IEEE Conference on Computer Communications (INFOCOM).

[15] Dardari D., Closas P., Djurić P.M. (2015). Indoor tracking: Theory, methods, and technologies. IEEE Trans. Veh. Technol..

[16] Belmonte-Hernández A., Hernández-Peñaloza G., Alvarez F., Conti G. (2017). Adaptive fingerprinting in multi-sensor fusion for accurate indoor tracking. IEEE Sens. J..

[17] Du Y., Yang D., Yang H., Xiu C. (2016). Flexible indoor localization and tracking system based on mobile phone. J. Netw. Comput. Appl..

[18] Xu W., Liu L., Zlatanova S., Penard W., Xiong Q. (2018). A pedestrian tracking algorithm using grid-based indoor model. Autom. Constr..

[19] Deng Z.A., Hu Y., Yu J., Na Z. (2015). Extended Kalman filter for real time indoor localization by fusing WiFi and smartphone inertial sensors. Micromachines.

[20] Belmonte-Hernández A., Hernández-Peñaloza G., Gutiérrez D.M., Álvarez F. (2019). SWiBluX: Multi-Sensor Deep Learning Fingerprint for precise real-time indoor tracking. IEEE Sens. J..

[21] Zhao Z., Braun T., Li Z., Neto A. (2018). A real-time robust indoor tracking system in smartphones. Comput. Commun..

[22] Jia R., Jin M., Zou H., Yesilata Y., Xie L., Spanos C. (2016). Mapsentinel: Can the knowledge of space use improve indoor tracking further?. Sensors.

[23] Wu C., Zhang F., Wang B., Liu K.R. (2019). EasiTrack: Decimeter-Level Indoor Tracking With Graph-Based Particle Filtering. IEEE Internet Things J..

[24] Marelli D., Fu M., Ninness B. (2015). Asymptotic Optimality of the Maximum-Likelihood Kalman Filter for Bayesian Tracking With Multiple Nonlinear Sensors. IEEE Trans. Signal Process..

[25] Wang W., Marelli D., Fu M. (2020). Fingerprinting-Based Indoor Localization Using Interpolated Preprocessed CSI Phases and Bayesian Tracking. Sensors.

[26] Thrun S. (2002). Probabilistic robotics. Commun. ACM.

[27] Arulampalam M.S., Maskell S., Gordon N., Clapp T. (2002). A tutorial on particle filters for online nonlinear/non-Gaussian Bayesian tracking. IEEE Trans. Signal Process..

[28] Wu K., Xiao J., Yi Y., Chen D., Luo X., Ni L.M. (2012). CSI-based indoor localization. IEEE Trans. Parallel Distrib. Syst..

[29] Wang X., Gao L., Mao S., Pandey S. DeepFi: Deep learning for indoor fingerprinting using channel state information. Proceedings of the 2015 IEEE Wireless Communications and Networking Conference (WCNC).

[30] Wang X., Gao L., Mao S. (2016). CSI phase fingerprinting for indoor localization with a deep learning approach. IEEE Internet Things J..

[31] Xie Y., Li Z., Li M. (2018). Precise power delay profiling with commodity Wi-Fi. IEEE Trans. Mob. Comput..

[32] Silverman B.W. (1986). Density Estimation for Statistics and Data Analysis.

[33] 3GPP (2019). TR38.855. Study on NR Positioning Support (Release 16).

[34] Devore J.L., Berk K.N. (2012). Modern Mathematical Statistics with Applications.

[35] Petersen K., Pedersen M. (2008). The Matrix Cookbook.

